# Differential Contributions of Inhibitory Subnetwork to Visual Cortical Modulations Identified *via* Computational Model of Working Memory

**DOI:** 10.3389/fncom.2021.632730

**Published:** 2021-05-20

**Authors:** William H. Nesse, Zahra Bahmani, Kelsey Clark, Behrad Noudoost

**Affiliations:** ^1^Department of Mathematics, University of Utah, Salt Lake City, UT, United States; ^2^Department of Biomedical Engineering, Tarbiat Modares University, Tehran, Iran; ^3^Department of Ophthalmology, University of Utah, Salt Lake City, UT, United States

**Keywords:** working memory, phase locking, local field oscillations, excitatory-inhibitory balance, visual responses

## Abstract

Extrastriate visual neurons show no firing rate change during a working memory (WM) task in the absence of sensory input, but both αβ oscillations and spike phase locking are enhanced, as is the gain of sensory responses. This lack of change in firing rate is at odds with many models of WM, or attentional modulation of sensory networks. In this article we devised a computational model in which this constellation of results can be accounted for via selective activation of inhibitory subnetworks by a top-down working memory signal. We confirmed the model prediction of selective inhibitory activation by segmenting cells in the experimental neural data into putative excitatory and inhibitory cells. We further found that this inhibitory activation plays a dual role in influencing excitatory cells: it both modulates the inhibitory tone of the network, which underlies the enhanced sensory gain, and also produces strong spike-phase entrainment to emergent network oscillations. Using a phase oscillator model we were able to show that inhibitory tone is principally modulated through inhibitory network gain saturation, while the phase-dependent efficacy of inhibitory currents drives the phase locking modulation. The dual contributions of the inhibitory subnetwork to oscillatory and non-oscillatory modulations of neural activity provides two distinct ways for WM to recruit sensory areas, and has relevance to theories of cortical communication.

## 1. Introduction

Top-down signals modulate responses to incoming sensory stimuli (Desimone and Duncan, 1995; Humphreys et al., 1998; Lee et al., 2005; Mitchell et al., 2007; Fries, 2009, 2015; Churchland et al., 2010; Bosman et al., 2012; Vinck et al., 2013; van Kerkoerle et al., 2014; Womelsdorf and Everling, 2015; Engel et al., 2016; Michalareas et al., 2016; Moore and Zirnsak, 2017), and have been explored in computational models (Brunel and Wang, 2001; Ardid et al., 2007; Lakatos et al., 2008; Kopell et al., 2011; Lee et al., 2013; Kanashiro et al., 2017). It has been shown that the firing rates of extrastriate cortical neurons are not modulated by working memory (WM) (Lee et al., 2005; Zaksas and Pasternak, 2006; Mendoza-Halliday et al., 2014). However, in the presence of a sensory signal, firing rates of neurons in these areas are enhanced when the stimulus in their receptive field is the target of top-down attention or WM (Moran and Desimone, 1985; Treue and Maunsell, 1996; Treue and Martínez Trujillo, 1999; Zhou and Desimone, 2011; Merrikhi et al., 2018). The goal of this study is to provide insight into the neural mechanisms giving rise to this non-linear interaction between sensory stimulus and top-down signal: to explain how extrastriate responses to sensory stimuli are modulated, without changes in baseline firing, to account for WM- and attention-induced changes in spiking behavior. Both attention and WM also induce oscillations in local field potentials (LFPs) (Fries et al., 2008; Siegel et al., 2008; Gregoriou et al., 2009; Liebe et al., 2012; Daume et al., 2017) and the timing of spikes relative to these oscillations (Lee et al., 2005; Vinck et al., 2013; Bahmani et al., 2018; Drebitz et al., 2018; Fiebelkorn and Kastner, 2020). These oscillations are believed to contribute to enhanced sensory processing (Bahmani et al., 2018). Thus, to achieve our goal, we developed a minimal dynamical model mimicking oscillatory behavior in the network and investigated how top-down and bottom-up signals interact to enhance representations of sensory information.

Previous work by our group established that extrastriate areas (such as V4 or MT) receive direct input reflecting the content of WM from prefrontal cortex (Merrikhi et al., 2018). In another recent article (Bahmani et al., 2018), we found that in the absence of any bottom-up sensory input, WM increased LFP αβ-band power, and neurons were more likely to fire at specific ranges of phase angles of the αβ-band LFP oscillation—phase locking. However, these changes were not accompanied by a change in the overall firing rate, although firing rate response gain was enhanced with the inclusion of sensory input at the receptive field (RF) location held in WM. Importantly, effect sizes depended on the degree of overlap between the remembered location and the visual neuron's RF, suggesting this phenomenon was restricted to a local network with common RF input.

To understand how local network mechanisms could produce the newly-observed WM effects in Bahmani et al. (2018), we simulated a conductance-based network model consisting of small-scale excitatory and inhibitory subpopulations. We employed a simple model capable of generating oscillations, without incorporating layer-specific subpopulations; this minimal model was sufficient to replicate the pattern of neural results and allow us to interrogate the relationship between oscillatory changes and spiking responses. We found that WM-dependent elevated activity in the inhibitory subnetwork produces emergent oscillatory behavior, and reproduces the observed the gain modulation and phase locking effects in excitatory neurons. This is consistent with established links between inhibitory neurons and attention (Mitchell et al., 2007; Kanashiro et al., 2017), and modeling studies linking inhibition to network oscillations (Van Vreeswijk et al., 1994; Chow et al., 1998; White et al., 1998; Brunel and Hakim, 1999; Whittington et al., 2000), like that of the ING-oscillation (Tiesinga and Sejnowski, 2009). Note that the dynamical origins of the persistent WM input were not studied here, instead we focus on the effects of this input on sensory networks (for the former, see for instance Wang, 2001). Our model predicted differential effects of WM input on firing rate and phase locking for excitatory vs. inhibitory neurons, and a re-analysis of the data from Bahmani et al. (2018) based on spike width (Mitchell et al., 2007) confirmed that firing rate is modulated only for putative inhibitory neurons, while phase locking is only modulated for the excitatory neurons.

To understand how the gain and phase locking effects emerged in the network model, we developed a spiking phase-oscillator model (Glass and Mackey, 1988) that accepts top-down, bottom-up, and oscillatory input generated from the network model. This phase model allowed the independent manipulation of two factors that affect network behavior. The first factor affects the inhibitory to excitatory balance due to inhibitory gain saturation, resulting in enhanced excitatory responses to bottom-up input (Kapfer et al., 2007; Vogels and Abbott, 2009; Kanashiro et al., 2017). The second factor relates to inhibition's effect on phase-coupling that shapes the trajectory to spiking in response to oscillating network input, and strongly affects spike phase locking. Our findings suggest that inhibitory subnetworks that are selectively activated by WM play a dual role in influencing excitatory cells, modulating both the sensory gain and phase locking to network oscillations. These WM-mediated changes underly two principal hypotheses for top-down control of interareal cortical communication (Diesmann et al., 1999; Womelsdorf et al., 2007; Kumar et al., 2008; Fries, 2015; Moldakarimov et al., 2015; Kanashiro et al., 2017; Hahn et al., 2019; Kohn et al., 2020), where both spike count, as well as the degree of spike synchronization within an oscillation cycle, each contribute to communication efficacy.

## 2. Methods

### 2.1. Experimental Methods and Neural Data Analysis

Two adult male rhesus monkeys (*Macaca mulatta*) were used in this study. All experimental procedures were in accordance with the National Institutes of Health Guide for the Care and Use of Laboratory Animals, the Society for Neuroscience Guidelines and Policies. The protocols for all experimental, surgical, and behavioral procedures were approved by the Montana State University Institutional Animal Care and Use Committee. All surgical procedures were carried out under Isoflourane anesthesia and strict aseptic conditions.

Cortical neurons in the middle temporal area (MT) of macaque monkeys were recorded during a memory guided saccade (MGS) task. Experimental techniques and details of the behavioral task are detailed in Bahmani et al. (2018); however we provide a brief sketch here. An MGS task trial consisted of a time to gain eye fixation on a marker at the center of a black screen, followed by a location cue presentation period at one of several peripheral locations, one of which was located within the RF of recorded MT neurons–the IN condition—or outside of the RFs—the OUT condition. After the cue disappeared, a 1-s delay period was presented in which no visual input was given. Following the delay period a saccade was initiated to the remembered location. Data from the delay period of correct trials were reanalyzed here to assess the influence of WM, either IN or OUT of the receptive field, on cell spiking, local fields, and phase locking.

Spike phase locking *SPL* is a quantity between zero and unity that measures the degree of spiking regularity with respect to an oscillating signal. Let ℓ(*t*) be the LFP signal and ℓ_αβ_(*t*) be the αβ bandpass filtered LFP. The angle argument (arg) of the analytic continuation of ℓ, via the Hilbert transform H, defines the oscillation phase ϕ(*t*) of the LFP signal:

(1) $$\begin{array}{l} \phi (t)=\arg\;(\ell_{\alpha \beta }(t)+iH[\ell_{\alpha \beta }(t)])\in (-\pi ,\pi ]. \end{array}$$

If *t*_*n*_ are the spike times in a simulation or recording session *n* = 1, 2, …, *N*, then ϕ_*n*_ = ϕ(*t*_*n*_) are the spike phases. The *SPL* for that session is then defined as

(2) $$\begin{array}{l} SPL=|\frac{1}{N}\sum_{n=1}^{N}\exp(i\phi_{n})|, \end{array}$$

Where $i=\sqrt{-1}$ is the imaginary unit and the |·| is the complex modulus. Therefore, the *SPL* is the mean vector strength of the of spike phase angle sample, which represents how concentrated the phases are around the mean phase.

The modulation index (*MI*) was calculated as follows: if *X*_*i*_ is a measured quantity (firing rate or SPL) in condition *i* of the experiment, then the *MI* of condition *i* relative to condition *j* is

(3) $$\begin{array}{r} MI=\frac{X_{i}-X_{j}}{X_{i}+X_{j}}. \end{array}$$

To identify differential modulation of narrow and broad wave forms subpopulations to SPL and firing rate modulations, we segmented the spike waveform data used in our prior study (Bahmani et al., 2018) with different segmenting cutoff points. We computed the *MI* for SPL as well as firing rate (FR) for each segmenting cutoff. We looked for cutoff values that exhibited strong levels of Wilcoxon Rank-Sum significance in the respective measures of SPL and firing rate.

### 2.2. Computational Network Model

We created a minimal model of a cortical circuit composed of recurrent connections between and within two subpopulations, each composed of *N* = 10 model neurons. One subpopulation consisted of inhibitory interneurons (i-cells), while the other consisted of excitatory (e-cell) model neurons. This minimal network model is formulated to describe dynamics of a local network in extrastriate visual cortex, such as V4 or MT comprising cells tuned to the same receptive field like those reported in prior studies from in our lab (Merrikhi et al., 2017; Bahmani et al., 2018).

In addition to the recurrent connections, the populations received tonic excitatory input currents labeled as “top-down” WM input and “bottom-up” sensory input. The key distinction between WM and sensory inputs is in the relative strength of that input onto the i-cell and e-cell populations. The WM input is more strongly weighted to the i-cell population, whereas the sensory input is weighted more to the e-cell population. The mixing of WM and sensory inputs was designed to produce a range of firing patterns through adjusting the relative activity of i- and e-cell populations.

Each neuron was modeled as a leaky integrate-and-fire (LIF) of membrane voltage *v*(*t*). When *v* < *v*_*T*_, the threshold voltage for firing, the voltage was modeled by the standard capacitative current balanced with synaptic and other membrane currents

(4) $$\begin{array}{r} \tau_{v}\frac{dv}{dt}=v_{m}-v+w_{ji}s_{i}\left(v_{i}-v\right)+w_{je}s_{e}\left(v_{e}-v\right)+I_{j}+\gamma , \end{array}$$

where *j* = *i, e* depending on the type of neuron. A spike occurs at time *t*_*k*_ when $\lim_{t\to t_{k}^{-}}v(t)=v_{T}$, and then the voltage is reset to the reset voltage $\lim_{t\to t_{k}^{+}}v(t)=v_{R}$. Note the membrane currents as written above are not true currents, but instead expressed in units of mV. In order of appearance from left to right, the currents are the capacitative current equal to the sum of a passive leak current *v*_*m*_−*v*, inhibitory *w*_*ji*_*s*_*i*_(*v*_*i*_−*v*) and excitatory synaptic currents *w*_*je*_*s*_*e*_(*v*_*e*_ − *v*), and extrinsic current *I*_*j*_ (comprising both top-down and bottom-up influences), and independent noise current γ, respectively.

The network was recurrently connected all-to-all with fixed-value synaptic weights *w*_*ee*_, *w*_*ii*_, *w*_*ie*_, and *w*_*ei*_ in (4) (see Table 1 for specific values). The time-dependent inhibitory and excitatory synapse variables *s*_*i*_(*t*) and *s*_*e*_(*t*), respectively, are governed by a standard two-variable system of DEs:

(5) $$\begin{array}{l} \tau_{j}\frac{d\eta }{dt}=\eta +\frac{1}{N}\sum_{k,j}\delta (t-t_{k}^{j}),\\ \tau_{j}\frac{ds_{j}}{dt}=-s_{j}+\eta , \end{array}$$

where $t_{k}^{j}$ are the spike times from the network from the *j*th population–*e* or *i*–and δ is the Dirac pulse functional. In the synaptic model (5), each *N* = 10 subpopulation exploits the linearity of the synaptic DE, in which all spikes in the subpopulation are summed together in the input to the η-equation. This summation means that each cell is self-coupled, however with *N* = 10 the effect of self-coupling is minimal.

**Table 1:** Parameter values for the LIF network model.

**Parameter**	**Value**	**Parameter**	**Value**
*v*_*T*_	−50 mV	*v*_*R*_	−60 mV
*v*_*e*_	0 mV	*v*_*i*_	−100 mV
τ_*v*_	10 ms	τ_*i*_	75 ms
τ_*e*_	5 ms	τ_*c*_	1000 ms
σ	0.05 mV	σ_*c*_	0.001 mV
*I*_0,*e*_	0.42 + 0.0089 (n − 1) mV	*I*_0,*i*_	0.28 + 0.0089 (n − 1) mV
α_*e*_	0.8 mV	α_*i*_	0.38 mV
β_*e*_	0.625 mV	β_*i*_	0.73 mV
*w*_*ee*_	0.002	*w*_*ii*_	0.02
*w*_*ie*_	0.04	*w*_*ei*_	0.02

The excitatory synapses were chosen to have a standard fast AMPA-type kinetics. The inhibitory synapses, we chose a timescale τ_*i*_ = 75 ms and reversal potential *V*_*i*_ = 100 mV consistent with a GABA_B_-type synapse. This choice enabled us to replicate the α-β-band (8–25 Hz) oscillation, that was observed to exhibit WM-dependent modulation in Bahmani et al. (2018), and is consistent with numerous modeling studies that identify inhibitory kinetics as the principal factor in determining oscillation period in reciprocally connected inhibitory networks (Van Vreeswijk et al., 1994; Chow et al., 1998; White et al., 1998; Brunel and Hakim, 1999; Whittington et al., 2000).

The input current *I*_*j*_ in (4) is composed of WM and sensory inputs: *I*_*j*_ = *I*_*j,m*_(*m*) + *I*_*j,s*_(*s*), where *m* and *s* are the memory and sensory control variables, respectively. The input current to each subpopulation is as follows

(6) $$\begin{array}{r} I_{j}=\alpha_{j}s+\beta_{j}m+I_{0,j}, \end{array}$$

where the coefficients α_*j*_ and β_*j*_ set the input gain of sensory- and memory-input currents to each *j*th subpopulation. The parameters *s* ranged over increments—24 in total—of the interval [0, 1], and *m* was set at 0 or 1 for memory OUT and IN conditions, respectively. Based on experimental evidence that top down inputs to extrastriate areas preferentially activate inhibitory cells (Mitchell et al., 2007), we have set β_*e*_ < β_*i*_ and α_*e*_ > α_*i*_, so that the WM input elicits greater gain for i-cells, while e-cells have greater current gain to bottom-up input *s*. The offset *I*_0, *j*_ was set over a range of values to introduce heterogeneity to each cell in each subpopulation, as well as to ensure that e-cell population average baseline firing in the absence of bottom-up stimulus *s* = 0 is near 4–5 Hz for each condition. Specifically, we express *I*_0, *j*_ as

(7) $$\begin{array}{r} I_{0,j}=I_{base,j}+\Delta I_{base,j}\left(n-1\right), \end{array}$$

for *n* = 1, 2, 3, …, *N* (see Table 1).

The time-dependent noise current γ(*t*) to each cell is independent of all other cells, and is comprised of both uncorrelated Gaussian white noise ξ_1_(*t*) and Ornstein-Uhlenbeck correlated noise ζ(*t*)

(8) $$\begin{array}{r} \gamma \left(t\right)=\sigma_{w}\xi_{1}\left(t\right)+\zeta \left(t\right), \end{array}$$

(9) $$\begin{array}{r} \tau_{c}\frac{d\zeta }{dt}=-\zeta +\tau_{c}\sigma_{c}\xi_{2}\left(t\right), \end{array}$$

where ξ_2_(*t*) is another independent realization of Gaussian white noise. Ito stochastic integration dictates that both ξ_1_(*t*)*dt* and ξ_2_(*t*)*dt* are Wiener process differentials (Gardiner, 2009). The noise level was tuned to be large enough so that cells in the network exhibited smooth firing rate changes in response to input changes rather than stair-step jumps due to extreme phase locking (Chacron et al., 2000; Nesse et al., 2007).

The parameter settings for the model are listed in Table 1.

#### 2.2.1. LFP Proxy

The local field potential (LFP) proxy was composed of a linear combination of all excitatory and inhibitory currents in the network, similar to the approach detailed in Mazzoni et al. (2015). In our realization of the LFP proxy, we multiplied by a voltage factor of equal to the near rest potential *v* = −55 Mv, minus the reversal potentials for each type of synapse to properly weight the relative contribution to the return current LFP:

(10) $$\begin{array}{l} LFP_{proxy}(t)=(w_{ee}+w_{ei})(-55-0)s_{e}(t)\\ \;+(w_{ee}+w_{ei})(-55+100)s_{i}(t) \end{array}$$

#### 2.2.2. Phase Oscillator Model

Essential features of LIF model neuron *v*(*t*) oscillatory dynamics can be described by a relatively simple phase oscillator model. The phase model describes a cell in terms of an angle quantity θ(*t*) that passes through from zero to unity and so on to zero again in each oscillation cycle, but the phase can also be perturbed through synaptic inputs (Glass and Mackey, 1988). We consider this a “toy model” that captures only essential behavior of interest in this study and only resembles the qualitative behaviors observed in the network model. The simplified phase model is a feed forward only single cell model; and the advantage afforded by this approach is that we are able to isolate and independently manipulate factors that we could not study within the network model in an independent manner. A great deal of work in phase coupled oscillator theory aims for rigorous reduction of higher-dimensional dynamical models into lower-dimensional phase-based models, particularly in the weak-coupling regime (Kuramoto, 1984; Hoppensteadt and Eugene M. Izhikevich, 1997; Brown et al., 2004), and extensions into the strong coupling regime (Cui et al., 2009; Castejón et al., 2013; Wedgwood et al., 2013; Cannon and Kopell, 2015). In this article we approach the phase model not as a rigorous reduction of our conductance-based network model; but simply from a phenomenological perspective.

The simple phase model we use is given by the following stochastic differential equation

(11) $$\begin{array}{r} \frac{d\theta }{dt}=\omega +R\left(\theta \right)s_{LFP}\left(t\right)+\chi \left(t\right), \end{array}$$

where θ ∈ [0, 1) describes the state of an oscillating neuron, ω is the intrinsic spike rate, *R*(θ) is the phase response function (Brown et al., 2004), *s*_*LFP*_(*t*) is the oscillating input, and χ(*t*) is a noise term. The input *s*_*LFP*_(*t*) is the LFP (synaptic conductance) that was recorded from the conductance-based network simulations. The phase model spikes at a rate ω for a given fixed baseline input that represents the effect of bottom-up and top-down input. Whenever θ → 1^−^, the phase angle resets to zero thereafter, and so on to spike on the next cycle.

If unperturbed by any inputs, the oscillator cell fires with rate ω. Oscillator spiking is determined by the confluence of top-down and bottom-up input represented by the parameter ω, and the non-linear phase-dependent effect of network input via the *R*(θ)*s*_*LFP*_(*t*) term. The phase response function represents this phase-dependent modulation of input that produces this variable delay. In our model, *R*(θ) is given by a negatively sloped linear function

(12) $$\begin{array}{r} R\left(\theta \right)=m\theta +b, \end{array}$$

where *m* < 0 that determines the efficacy of input in this non-linear phase-dependent manner. Note that in the phase model, this linear phase response function (12) is *non-linear* in the phase model DE (11), because on the periodic domain θ ∈ [0, 1) due to the jump discontinuity at 1.

The negatively-sloped linear phase response function (12) is a reasonable, but simplified representation LIF model coupling. The negative slope *m* of (12) represents the similar voltage-dependence of the inhibitory synaptic current in the LIF model of Equation (4). In the LIF model, increasing voltage nearer to spike threshold increases inhibitory synaptic efficacy. Similarly, increasing phase nearer to spike threshold increases phase delay due to the negative phase-dependence in (12).

The noise term χ in (11) is an additive noise signal consisting of two components χ(*t*) = σ_θ_η(*t*) + ν(*t*), where σ_θ_η(*t*)*dt* is the Wiener process with standard deviation parameter σ_θ_ and ν(*t*) is an Ornstein-Uhlenbeck process defined by

(13) $$\begin{array}{r} \tau_{c}\frac{d\nu }{dt}=-\nu +\tau_{c}\sigma_{\nu }\xi_{3}\left(t\right), \end{array}$$

where ξ_3_(*t*)*dt* is another Wiener process. The phase model parameters are listed in Table 2.

**Table 2:** Parameter values for the phase oscillator model.

**Parameter**	**Value**	**Parameter**	**Value**
*m*	−0.058	*b*	−0.0325
*M*	−0.075	ω_0,*IN*_	0.0985
ω_0,*OUT*_	0.0035	ω_0,*NPC,IN*_	0.096
ω_0,*NPC,OUT*_	0.0064	ω_0,*INeq,NPC,IN*_	0.096
ω_0,*INeq,NPC,OUT*_	0.097	ω_0,*INeq,OUT*_	0.0064
Δω	0.126	Δω_*n*_	0.006 + 0.0013 (n − 1), n=1…10

**Computation**: All simulations of the stochastic DEs were computed in custom built MATLAB code (Natick, MA) using a first-order explicit stochastic Euler's method. We chose a time step Δ*t* = 0.0305 ms, which was used in order that 1-s simulation time intervals possessed a power of two—2^15^—number of points enabling efficient computation of the fast Fourier transform. Source code will be made available upon request from the corresponding author.

## 3. Results

Our goal was to develop a computational network model of extrastriate neurons sharing the same receptive field (likely corresponding to a cortical column) that combined top-down WM and bottom-up sensory signals, and captured the following four key aspects of the neuronal responses observed during a WM task (Bahmani et al., 2018). First, in the absence of sensory input, the WM signal alone does not modulate excitatory unit firing rates, which we term the Parity aspect. Second, despite this lack of excitatory unit firing rate modulation, a WM signal alone modulates oscillatory power in the αβ-band of the LFP, but not in other bands, termed the Power aspect. Third, WM modulates the timing of spikes relative to the αβ oscillation (*SPL*), the Phase aspect. Finally, in the presence of bottom-up sensory input, a WM signal increases the gain of sensory responses, termed the Gain aspect.

**Figure 1:**
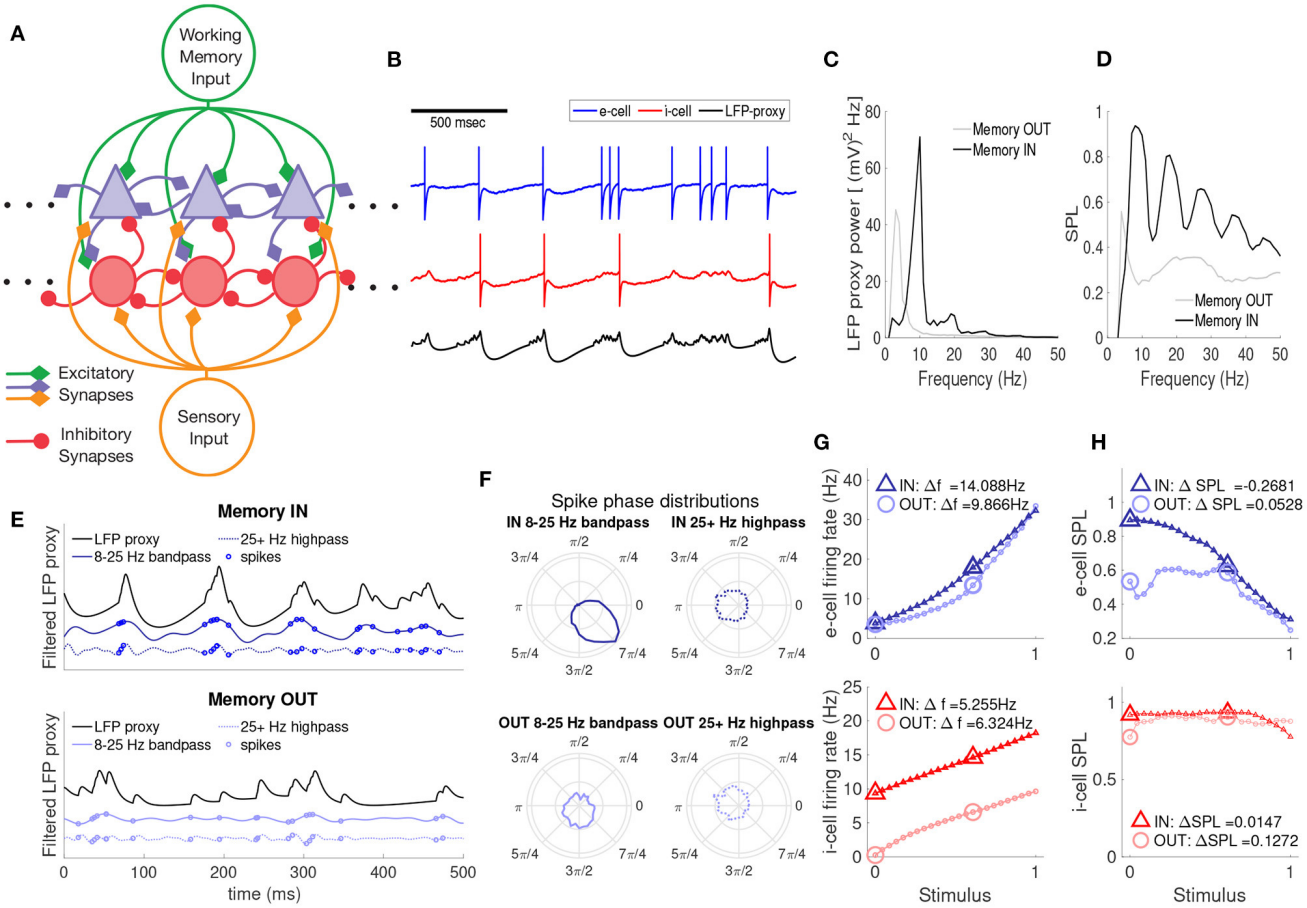
A recurrently connected spiking network model yields WM-dependent oscillations and spike timing, WM-independent baseline firing rates, and WM-dependent gain in sensory responses. **(A)** A schematic of the e- and i-cell network (blue triangles and red circles, respectively) with excitatory top-down (green) and bottom-up inputs (orange). **(B)** Example spike traces for an e-cell (blue), i-cell (red), and LFP proxy (black). Spike appearance modified for visual clarity. **(C)** Power spectrum of the LFP proxy for the memory IN and OUT conditions, in the absence of bottom-up input. **(D)** Spike phase locking (SPL) of all cells in the network to the phase of the bandpass filtered LFP oscillations across frequencies, for memory IN and OUT. **(E)** An example of the LFP proxy (black), αβ-band oscillations (solid blue), and high frequency oscillations (dashed blue) over time, along with the timing of spikes (blue circles), for memory IN (top) and OUT (bottom). **(F)** Spike-phase distributions (polar plots) for the αβ (left) and γ-band (right) LFP in the IN (top) and OUT conditions (bottom). **(G)** Mean firing rates of e-cells (top) and i-cells (bottom) as a function of bottom-up input strength, for memory IN (triangles) and OUT (circles). Here and in H, larger icons indicate the values used to compute the change in FR/SPL. **(H)** SPL for e-cells (top) and i-cells (bottom) as a function of bottom-up input strength.

Our model reproduces these four aspects of the experimental data (Parity, Power, Phase, and Gain), as shown in Figure 1. The basic network model structure, along with example spiking and LFP-proxy output, are shown in Figures 1A,B. The neuronal network model consisted of *N* = 20 leaky integrate and fire (LIF) cells, 10 excitatory and 10 inhibitory, as depicted in Figure 1A (not all connections shown, see section 2.2). Inputs to the network consist of top-down working memory input (green lines) and bottom-up sensory input (orange lines). The two network inputs are both excitatory and non-oscillatory, but differ in the relative strength of their connections to the e- and i-cells; e-cells are more strongly driven by bottom-up inputs, whereas i-cells are more sensitive to top-down inputs, as suggested from prior experimental work (Mitchell et al., 2007). Example voltage traces of e-cells (blue) and i-cells (red), including spikes, are shown in Figure 1B. A proxy of the local field potential (LFP-proxy) was also generated from the network (black), using a weighted sum of all synaptic conductances in the network (Mazzoni et al., 2015). Despite the lack of oscillatory input to the network, there are quasi-regular oscillatory fluctuations in the LFP-proxy, indicative of the emergent rhythmic behavior of the network, similar to prior computational studies involving reciprocal i-cell connections (Van Vreeswijk et al., 1994; Brunel and Hakim, 1999; Whittington et al., 2000; Brunel and Wang, 2001). Thus, this simple network architecture is able to generate oscillatory activity, which we can compare to the experimental LFP data under various combinations of top-down and bottom-up input.

First we examined the firing rate and LFP power spectrum of the model with and without top-down input, in the absence of sensory input; in the experimental data, top-down input alone altered αβ LFP power but not firing rates. The network conditions with and without top-down input are referred to as memory IN and OUT, respectively (corresponding to the experimental conditions when the remembered location was in the neuron's RF or outside it). We examined the behavior of the network for memory IN and OUT in the absence of bottom-up sensory inputs. In the absence of top-down input (OUT condition), the e-cells spiked at low rates (mean near 5 Hz, see B) and there were quasi oscillatory patterns present in the LFP-proxy that were chiefly in the θ-band (4–7 Hz, Figure 1C). During the memory IN condition, e-cell firing rates were similarly low, but the LFP-proxy power spectrum peaked in the αβ-band (8–25 Hz), as well as integer-multiples (harmonics) of that frequency. This top-down modulation of αβ LFP power but not firing rates replicates the Parity and Power aspects from the experimental data discussed above (Bahmani et al., 2018).

We next asked how spikes from cells in the network were timed relative to the LFP proxy oscillation; in the experimental data, top-down input increased SPL in the αβ band. The IN condition shows strong phase locking of all multi-unit spikes in the network, in the α-band (10–12 Hz) and its harmonics, while the OUT condition shows much weaker phase locking over the same frequency windows (Figure 1D). The strong phase locking of IN-condition spikes to the αβ component of the LFP is illustrated in Figure 1E (top): spikes are preferentially emitted at or just before the crests of the αβ LFP proxy. In contrast, the OUT condition and the >25Hz LFP showed less systematic relationships between spike timing and oscillations. That is, only in the αβ-band and the memory IN condition was there a strong relationship between the spike times and the phase of the LFP-proxy, as shown by the spike phase distributions in Figure 1F. These WM modulations of spike timing relative to local oscillations are consistent with the Phase aspect of Bahmani et al. (2018).

To test whether the network model showed greater sensory responses in the presence of top-down input, as seen in the experimental data, we examined the behavior of the network when adding bottom-up input of varying strength, with or without a top-down signal. The top-down input was excitatory, but tuned so that the e-cells in the network exhibited equal firing rates between the IN and OUT conditions. The mean firing rates of e-cells of the network (Figure 1G top, blue) were therefore equivalent for the IN and OUT conditions when no bottom-up stimulus was present (zero on the abscissa). We also performed simulations with a bottom-up stimulus of increasing strength; over a broad range of bottom-up input strengths, e-cells in the IN condition exhibited elevated firing rates relative to OUT (Figure 1G), reproducing the Gain aspect of Bahmani et al. (2018). In contrast, i-cell firing rates (Figure 1G bottom, red) were greater for the IN condition regardless of the strength of bottom-up input.

This model's change of tone in the inhibitory subnetwork appeared to be the key factor in generating both the oscillatory (Power and Phase) and non-oscillatory (Parity and Gain) aspects of the e-cell responses. The model suggests that the content of top-down WM-input should be reflected in the spiking activity of the inhibitory subnetwork, even in the absence of bottom-up sensory input. However, in the experimental data, we did not separately examine the responses of excitatory and inhibitory neurons, and so cannot say whether inhibitory neurons reflected the content of WM in the absence of sensory input. The model results motivated us to divide our data set into putative excitatory (broad spiking) and putative inhibitory (narrow spiking) units, and investigate whether inhibitory neurons exhibit WM-dependent changes in firing rate.

### 3.1. Distinct WM Modulation of Excitatory vs. Inhibitory Extrastriate Neurons

The model network simulations were set up so that WM input strongly modulated inhibitory neuron firing while not affecting excitatory firing. That is, in the absence of bottom-up input, e-cell firing rates were the same in the IN and OUT conditions, whereas i-cell firing rates were higher for the IN condition. Conversely, in the absence of bottom-up input, e-cell SPL was elevated for IN vs. OUT, while i-cell SPL was essentially unchanged. The e-cells in our model network replicate the results in Bahmani et al. (2018), but in this study we did not segment cells into e- or i-categories; however, past studies have observed elevated recruitment of inhibitory cells during periods of attention (Mitchell et al., 2007).

**Figure 2:**
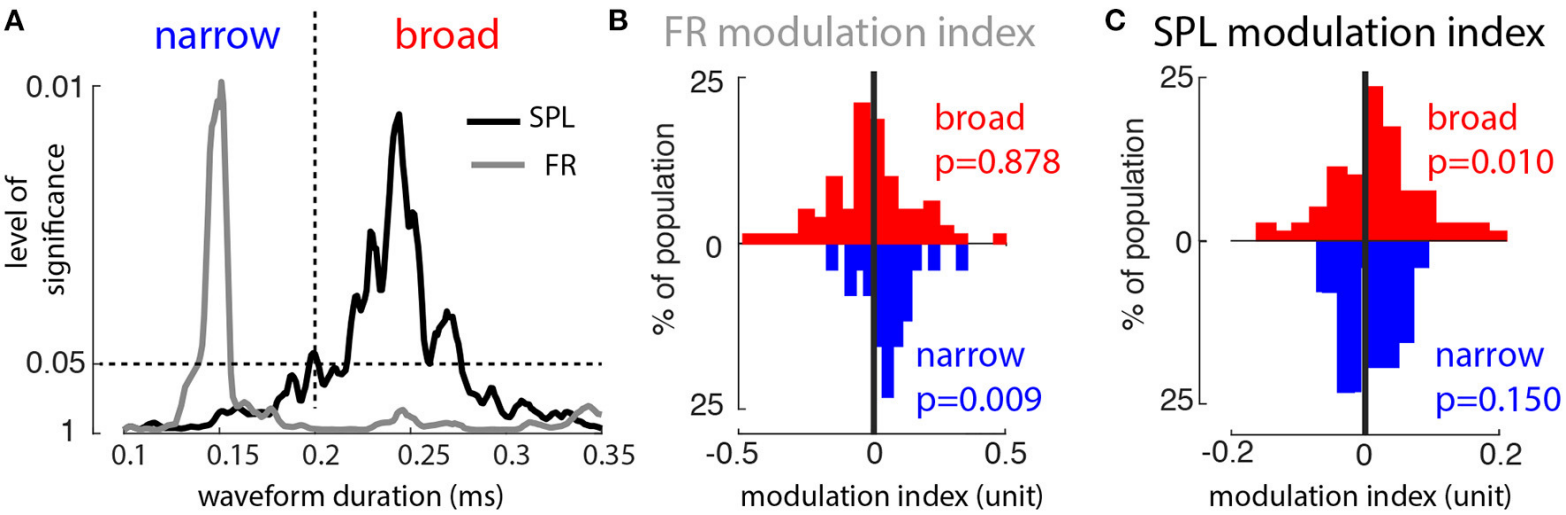
Broad- and narrow-waveform spikes are differently modulated by top-down input. **(A)** *P*-value for significance of population modulation (IN vs. OUT) of firing rate (gray) and SPL (black), as a function of waveform width (ordinate scale is 1/*P*). Vertical dashed line indicates width used to divide narrow and broad waveforms. **(B)** Distribution of firing rate modulation index values (IN vs. OUT) across the populations of broad (red) and narrow (blue) waveforms. **(C)** Distribution of SPL modulation index values (IN vs. OUT) across the populations of broad (red) and narrow (blue) waveforms.

To reconcile our model results with our prior experimental results, we reexamined that data set to determine if there were differing e- and i-cell modulations firing rate and SPL modulation due to WM. We divided the recorded MT spike waveforms into putative excitatory and inhibitory groups based on temporal widths of action potential waveforms, where narrow and broad waveforms correspond primarily to inhibitory and excitatory cells, respectively (Mitchell et al., 2007). To find a cutoff point between narrow and wide waveforms, we computed the sign-rank significance level of the modulation index (MI) for firing rate and SPL, IN vs. OUT (see section 2.1). For very narrow waveforms, i.e., putative inhibitory cells, there was a substantial firing rate modulation by WM in the absence of sensory input. In contrast, the MI for SPL was strongly significant only for broad waveforms (putative excitatory cells). From this analysis a partition of 0.2 ms spike width was chosen to distinguish narrow and broad spiking neurons (*n* = 26 narrow and *n* = 81 broad spiking neurons; Figure 2A). Putative excitatory cells showed no significant firing rate modulation, whereas inhibitory cells showed higher firing rates for the memory IN condition (Figure 2B, Wilcoxon sign-rank tests, *p* = 0.878 and *p* = 0.009, respectively). Putative excitatory cells showed enhanced αβ SPL during memory IN, whereas inhibitory cells showed no significant difference in αβ SPL between the IN and OUT conditions (Figure 2C; Wilcoxon sign-rank tests, *p* = 0.010 and *p* = 0.15, respectively). This new analysis of the previous dataset matched the model's predictions: WM modulation of firing rate only for inhibitory neurons, and SPL only for excitatory neurons.

The model replicated our prior experimental findings through WM-dependent changes in the inhibitory tone of the network, and upon reanalysis of our data we further confirmed that WM-dependent changes in inhibitory tone are present, which is consistent with studies of the effects of attention by other groups (Mitchell et al., 2007). These findings support a similar cellular- and circuit-level mechanism underlying both WM and attention, further establishing the interdependence of these two cognitive processes (van Kerkoerle et al., 2017).

### 3.2. Stimulus and Frequency Dependence of LFP, Spike Variability, and SPL

The model was designed to reproduce the four key experimental findings discussed above. Its success in predicting the differences in excitatory and inhibitory modulations not previously examined in the data suggests that it can provide further mechanistic insights as well. The experimental data had limitations, for example, we were not able systematically examine the influence of changes in bottom-up input on the network behavior. Using the computational model enables us to predict the behavior of the network in response to changes in bottom-up input, and compare the results to previous literature.

**Figure 3:**
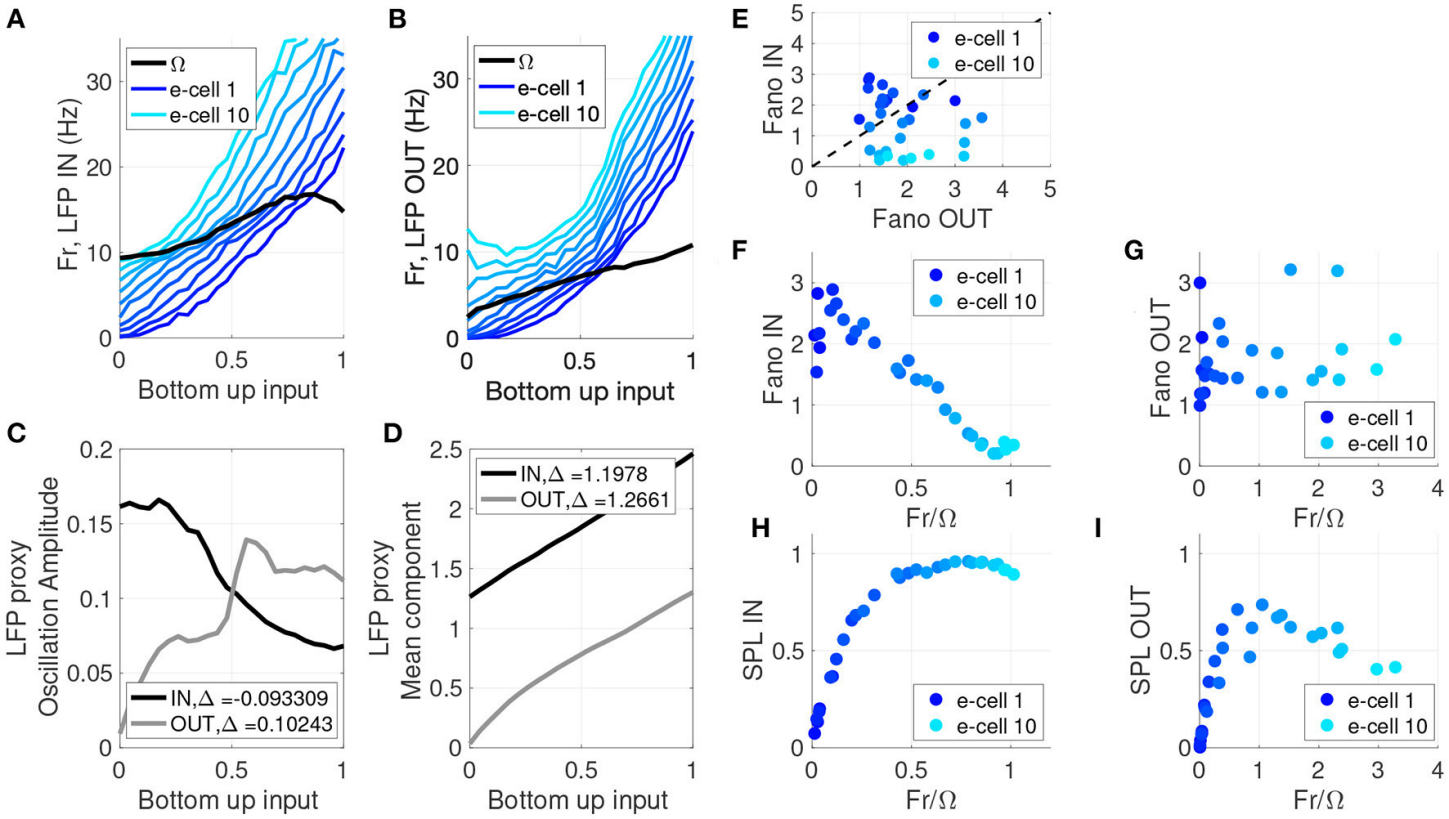
LFP proxy, cell firing rate, SPL, and spike variability show stronger frequency dependence for IN relative to OUT. **(A)** Frequency with peak LFP power (black) and the firing rate of individual cells (blue) as a function of bottom-up input strength, for the memory IN condition. Throughout this figure the model units are ranked based on intrinsic baseline firing rate, with dark blue to light blue indicating low to high firing rate model neurons. **(B)** Frequency with peak LFP power (black) and the firing rate of individual cells (blue) as a function of bottom-up input strength, for the memory OUT condition. **(C)** Amplitude of the LFP proxy oscillation at the peak frequency, as a function of bottom-up input strength, for memory IN (black) and OUT (gray). **(D)** Mean component of the LFP proxy oscillation at the peak frequency, as a function of bottom-up input strength, for memory IN (black) and OUT (gray). **(E)** Fano Factor for IN vs. OUT for 10 e-cells and three bottom-up input levels. **(F)** Fano Factor for memory IN as a function of the ratio between the peak LFP frequency and the cell's firing rate, for 10 e-cells and three bottom-up input levels. **(G)** Fano Factor for memory OUT as a function of the ratio between the peak LFP frequency and the cell's firing rate, for 10 e-cells and three bottom-up input levels. **(H)** SPL for memory IN as a function of the ratio between the peak LFP frequency and the cell's firing rate, for 10 e-cells and three bottom-up input levels. **(I)** SPL for memory OUT as a function of the ratio between the peak LFP frequency and the cell's firing rate, for 10 e-cells and three bottom-up input levels.

By examining the network model response to bottom up input, for both the IN and OUT conditions, we can compare the model results to other identified signatures of WM and attention in the literature. We found in our model that oscillation frequency and power of the network LFP exhibited input-dependence that was consistent with prior studies (Bosman et al., 2012; Roberts et al., 2013). The model showed the frequency with the most power in the LFP proxy (Ω) increased with increasing bottom-up input. In the IN condition, the frequency Ω went from the α-range (10 Hz), up to the mid-β-range (16 Hz) as the bottom-up input increased (Figure 3A). This increase in peak LFP frequency is consistent with prior experimental findings (Roberts et al., 2013) and modeling studies (Chow et al., 1998; Whittington et al., 2000; Brunel and Wang, 2003). In the memory OUT condition similar increases in Ω as a function of bottom-up input occurred, except Ω progressed from the θ to low-α band (4 Hz up to 10 Hz) (Figure 3B). This resembles the reported effect of top-down input in the form of attention, which increases the peak frequency of oscillations; however in that case the shift occurred within the gamma band (Bosman et al., 2012). The oscillation amplitude at the Ω frequency (i.e., the dominant power spectrum mode of the LFP) also shows a clear dependence on the top-down WM input signal (Figure 3C). The IN condition has strong αβ oscillations in the absence of bottom-up input, but their amplitude declines as bottom-up input increases. In contrast, the OUT condition starts at near zero amplitude, first growing and then declining with increased bottom-up input. These patterns of oscillation amplitude as a function of bottom-up input are a novel prediction that has yet to be addressed in the literature.

**Figure 4:**
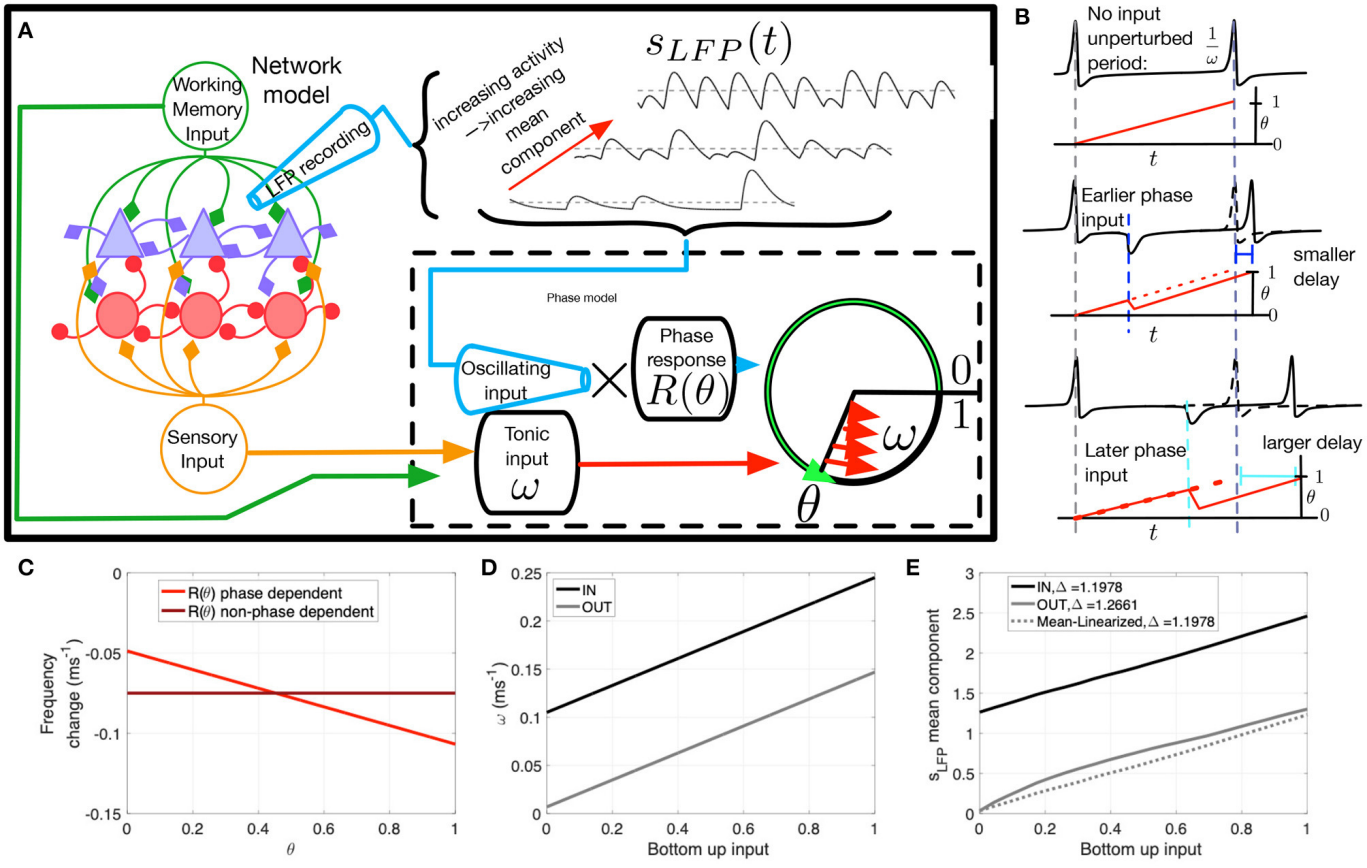
The phase oscillator model and parameters. **(A)** Schematic of the network model. The LFP proxy *s*_*LFP*_(*t*), recorded from the network, is inputted to the phase model. The mean or baseline level of the LFP can vary (dashed). The phase model receives inhibitory input from the LFP proxy signal combined with intrinsic excitability through ω, the proxy of the top-down and bottom-up input in the network model. **(B)** Spike delay as a function of input timing. ω dictates unperturbed spiking frequency (top). If input is timed early in the oscillatory cycle there is a moderate delay in spiking (middle); however a later input produces a longer delay (bottom), resulting from greater efficacy of inhibition when a cell is more depolarized. **(C)** Change in firing rate as a function of input phase. The phase response curve *R*(θ) (bright red) is negatively sloped to produce the variable delay effect depicted in D. A flat phase response curve *R* = *M* (dark red) was used to test the consequences of this phase dependence. **(D)** ω values as a function of bottom-up input for IN (black) and OUT (gray). **(E)** The LFP mean component *mean*(*s*_*LFP*_(*t*)) as a function of bottom-up input for IN (black) and OUT (gray). The mean-linearized version (dashed) was used to test the consequences of inhibitory saturation.

**Figure 5:**
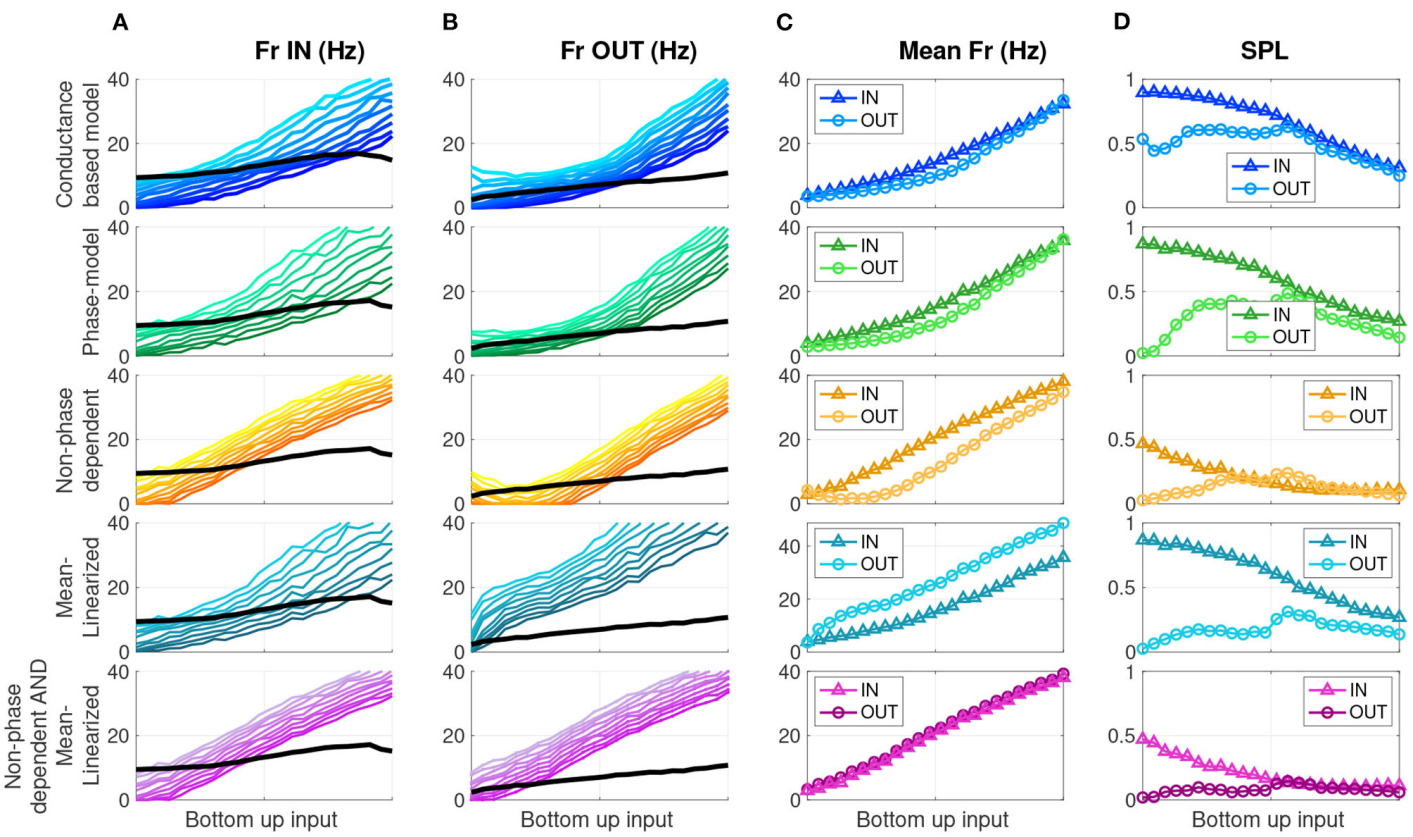
Modified phase oscillator models demonstrate distinct drivers of firing rate and SPL modulation. Each row shows a different model, labeled on the left. **(A)** Firing rate for 10 cells/simulations (colors) and the Ω frequency (black) as a function of bottom-up input for the memory IN condition. **(B)** Same for memory OUT. **(C)** Mean firing rate as a function of bottom-up input, for memory IN and OUT. **(D)** Mean SPL as a function of bottom-up input, for memory IN and OUT.

Beyond the oscillatory aspects of the network, we also examined non-oscillatory properties of the network in terms of the influence of WM on its input-output relationship. We considered the LFP mean component (i.e., the DC component, see section 2.2.1) as a measure of average network output, and the slope of the input-output curve as a measure of the synaptic efficacy. The LFP mean component exhibits monotonic growth with increased bottom-up input, but the degree of growth (synaptic efficacy) differs between the IN and OUT conditions (Figure 3D). In the OUT condition the mean starts near zero, and the growth is slightly non-linear and saturating in nature. This non-linearity has on average an elevated slope relative to the IN condition, and this yields a slightly greater mean change over the bottom-up input range: Δ = 1.266 OUT vs. Δ = 1.198 IN. There is a 3.6% greater overall mean change for OUT relative to IN, meaning the OUT condition has a greater change in inhibitory activity (compared to the IN condition) for the same change in bottom-up excitatory input. This difference in inhibition may account for the comparatively smaller firing rates observed in the OUT condition in response to bottom-up input (see Figure 1G and Vogels and Abbott, 2009; Kanashiro et al., 2017; Merrikhi et al., 2017). Thus, the non-oscillatory component of the network behavior (i.e., WM-dependent change in response gain) seems to depend on a separate mechanism from its oscillatory behaviors, a hypothesis that we will directly test in Figures 4, 5.

Both attention and WM have been shown to modulate the variability of neuronal responses (Fano factor) (Mitchell et al., 2007; Steinmetz and Moore, 2010; Merrikhi et al., 2017). As shown Figure 3E, we also observed a reduction of Fano factor for IN relative to OUT (OUT − IN = 0.426, *p* = 0.0439, one-sided paired Wilcoxon sign-rank). Our analysis of Fano and SPL in Figures 3E–I is limited to data generated when bottom-up input was low. Changes in Fano factor have been speculatively linked to changes in oscillations (Mitchell et al., 2007). Assessing the Fano values as a function of *Fr*/Ω in Figure 3F, we observed that the e-cells that exhibited the most substantial reductions in Fano factor have firing rates close to the LFP proxy peak frequency (*Fr* ~ Ω). This pattern did not hold for the OUT condition (Figure 3G). Note that *Fr*/Ω ~ 1 means that a model cell generates about one spike per oscillation cycle. As many theoretical studies have identified, phase locking is stronger when there are integer frequency ratios *Fr*/Ω, suggested by regions of stability (i.e., Arnold tongues) in phase oscillator models (Glass and Mackey, 1988; Shimokawa et al., 2000; Nesse and Clark, 2010). Therefore, as *Fr*/Ω values approach 1 in Figure 3F, a greater SPL is expected to accompany the reduced Fano factor. Indeed, in Figures 3H,I we directly assessed SPL as a function of *Fr*/Ω and confirmed that this reduced Fano factor is associated with greater SPL in the IN condition. Thus, both reductions in Fano factor and increases in SPL are more prominent in neurons with firing rates near that of the network oscillation frequency.

### 3.3. Assessing Mechanisms Underlying Changes in Spike Rate and Timing With the Phase Oscillator Model

Our network model of e- and i-cells receiving a top-down WM signal replicated both the oscillatory (Power and Phase) and non-oscillatory (Parity and Gain) aspects of the neural data. The degree to which these oscillatory and non-oscillatory aspects share overlapping mechanisms is not clear. Further insight into how the various modulations co-emerge requires a paradigm in which we can independently manipulate cellular- and network-level components of the system to test their effect on spiking behavior. The needed paradigm is phase coupled oscillator theory, which describes how cell and network oscillatory phase interactions influence the input-output relationship and phase locking behavior of individual cells (Glass and Mackey, 1988). Here, we created a series of phase oscillator models, one for each excitatory cell in the network model, in both top-down and bottom-up input conditions, and receiving oscillatory synaptic input (Figure 4A). The phase models were not dynamically coupled together in a network, but each cell received the same pre-recorded network model input to simulate the effect of network oscillations. The phase models differed in baseline excitability to mimic the cell heterogeneity found in the network model cells.

This simplified phase oscillator model can recreate the four aspects of the network model behavior, with the added benefit of allowing us to modify features of both the input as well as input coupling, in order to selectively modulate the oscillatory and non-oscillatory aspects of the e-cell response. Note our analysis focuses on identifying the factors relevant for controlling the Gain and Phase aspects, which are specific to the excitatory cells we wish to describe with the phase oscillator model. The phase models were all subject to the Parity constraint, which was achieved by parameter tuning to equalize average firing rate between the IN and OUT conditions when bottom-up input was low. The Power aspect was preserved in the oscillations generated by the full network model, which served as pre-recorded input to each phase model.

Each phase model's state is defined by a phase angle variable θ(*t*) which cycles from zero to unity as a function of its inputs, with spikes occurring when the phase angle reaches unity. Each phase model's spiking is determined by the combination of two inputs: an excitatory intrinsic spike frequency ω, which is a proxy of the sum of bottom-up and top-down tonic input in the network model, and an inhibitory oscillating input *s*_*LFP*_(*t*) recorded from the network model (Figure 4A). Note that *s*_*LFP*_(*t*) can be broken down into a mean (non-oscillating) component (dashed lines Figure 4A) that exhibits varying baseline levels, and an oscillating component, both of which depend on network activity (see Figures 3A–D).

The two inputs to the model, ω and *s*_*LFP*_(*t*), differ in that ω determines a tonic baseline rate of spiking, while the effect of the oscillating network input *s*_*LFP*_(*t*), owing to it being dominated by inhibitory synaptic currents, depends strongly on the depolarization state of the cell. This state-dependence translates to the phase model as a phase-dependent effect: the efficacy of input is modulated by a multiplicative factor *R*(θ)—the phase response function—that depends on the phase θ of the post-synaptic cell (dashed box in Figure 4A). Note that *s*_*LFP*_(*t*) input is positive-valued (see Equation 10) but has an inhibitory effect resulting in a delayed spike because the phase response function *R*(θ) is negative-valued.

The phase-dependent coupling in the model alters the timing of spikes as a function of the timing of an input relative to the previous spike. This is shown schematically in Figure 4B. In the absence of inputs, the cell fires at frequency ω (top subpanel). If a transient input is given early in the cell's oscillation cycle, then there is a moderate delay in firing (or possibly an advance in some cases; see Nesse and Clark, 2010, for example), but the same input later in the cycle will produce a longer delay (middle and bottom panels). The phase-dependent influence of input on the frequency of neuronal firing is illustrated in Figure 4C. Without phase dependence (dark red line), a pulse input would generate the same delay (−0.075 ms^−1^ frequency change), regardless of when it arrives. With the phase-response function (light red), the phase at which the pulse input arrives influences the change in the frequency of neural firing. The inputs from the network model to the phase oscillator model are shown in Figures 4D,E. The tonic input reflects a linear combination of bottom-up and top-down input. The mean component of the oscillatory input, however, shows a non-linear response to bottom-up input in the OUT condition. Thus, the change in the LFP mean component (IN-OUT) is greater at higher bottom up input, presumably a mechanism for greater gain in the IN condition. The advantage of the phase model is the ability to selectively modify its components: the mean linearized version of the model removes this non-linearity. Alternatively, as shown in Figure 4C, we are also able to remove phase-dependence of the phase model. Our hypothesis is that the mean component non-linearity drives Gain modulation, while the phase-dependence is responsible for SPL modulation.

Theories of cortical communication have proposed that a local network's quantity of spikes as well as their degree of synchronization are independent factors that affect the signal efficacy or propagation (Diesmann et al., 1999; Womelsdorf et al., 2007; Kumar et al., 2008, 2010; Fries, 2015; Moldakarimov et al., 2015; Kanashiro et al., 2017; Hahn et al., 2019; Kohn et al., 2020). In the oscillatory context, these quantities can be measured, respectively, by the firing rate and the degree of SPL in each oscillation cycle. To test the effect of phase dependence and inhibitory tone on firing rate and SPL, we ran four experimental conditions of each phase model cell simulation for both IN and OUT conditions—the full model, and versions with the phase dependence removed and/or mean-linearized. As stated above, we simulated these models over a range of *N* = 10 intrinsic baseline firing rates (to approximate the different responses in the *N* = 10-e-cell network), and over a range of bottom-up input levels defined by the ω parameter (see Figure 4D), for long-time-window simulations, and computed average firing rate and SPL statistics. That is, there were 10 × 2 × 24 × 4 distinct phase models simulations, respectively, for the ten cells with varying baseline rates, a factor with two levels to reflect IN vs. OUT, 24 bottom-up input levels, and four versions of the phase model with or without phase dependence and mean-linearization (see section 2.2.2). Figure 5 shows model output for the network model (top row), the phase oscillator model (2nd row), the non-phase-dependent model (3rd row), the mean-linearized model (4th row), and finally, the non-phase-dependent and mean-linearized model (bottom row). Columns A and B show individual cell firing rates (color gradation indicating distinct cells) over the range of bottom-up input for the IN and OUT conditions, respectively. Column C shows the mean firing rate across the 10 cells. Column D shows the mean SPL across the 10 cells.

The phase oscillator simulations (green, 2nd row) closely replicate the results of the conductance-based network model output (blue, top row) over all measures. Next, we evaluate the role of phase dependence and mean saturation in WM-modulations of firing rate and SPL. Phase-dependent coupling *R*(θ) means that output depends on both the input *s*_*LFP*_(*t*) and the current phase of the oscillator through the phase response curve *R*(θ). When this phase dependence is eliminated (Figure 5 orange, 3rd row), the SPL measures are dramatically reduced in both the IN and OUT conditions relative to the full phase model. In contrast, the difference in firing rate for IN vs. OUT is preserved. That is, the Parity and Gain aspects were preserved, while SPL was markedly reduced for both IN and OUT conditions. When the slope of the OUT mean LFP component input is linearized (mean-linearized model), the IN and OUT mean firing rate curves invert (Figure 5 slate-blue, 4th row) relative to the full model, with a higher firing rate during OUT than IN. SPL is higher for IN than OUT across the range of bottom-up inputs. Here, Parity at low inputs is preserved but the Gain aspect is entirely inverted, while the SPL aspect is largely unperturbed. When both manipulations (Figure 5 purple, 5th row) are introduced together—non-phase-dependence and mean-linearization—the mean firing rates no longer exhibit any difference between IN and OUT for any level of bottom-up input. The SPL values are low relative to the default model (2nd row), similar to that of the non-phase-dependent model (3rd row). Therefore, the combined effect of both manipulations eliminates both the Gain and SPL aspects together. The four combinations of manipulations constitute a double-dissociation, where the Gain aspect is attributed, in large part, to the differences in the mean component of the input *s*_*LFP*_ to cells. Thus, firing rate modulation depended primarily on saturation of the inhibitory response. In contrast, SPL modulation was primarily determined by the phase-dependence of the excitatory cell's sensitivity to inhibitory input.

## 4. Discussion

Our network model provides mechanistic level insight into how top-down input can modulate oscillatory power, spike timing, and sensory gain, without changes in baseline firing rates, as recently observed in Bahmani et al. (2018). This mechanism involves WM-dependent modulation of the inhibitory subnetwork, which enhances both non-oscillatory mean activity and oscillatory dynamics of the network, which in turn affects excitatory units, modifying their sensory gain and spike timing.

The WM input to the excitatory and inhibitory subnetworks were tuned so that the elevated inhibitory activity fully offsets the WM input to excitatory units in the absence of sensory input, leaving the firing rate of excitatory units unchanged relative to when no WM input is present. This differential input strength is consistent with prior observations (Mitchell et al., 2007), and is confirmed upon reanalysis of our data here; however, we do not speculate about any specific connection architecture that delivers this input. This accounts for the Parity aspect of the experimental results. However, this offsetting influence of the inhibitory subnetwork on the excitatory subnetwork has a limit due to the lower gain of the i-cells' response, and with additional bottom-up sensory input on top of the WM input, the activity of the inhibitory subnetwork does not keep pace with the excitatory drive, yielding an enhanced excitatory network response. This saturation phenomenon accounts for the Gain aspect of our experimental findings. This type of response saturation has been proposed as a mechanism for attentional (or WM-mediated) enhancement of excitatory unit responses in previous studies (Kapfer et al., 2007; Vogels and Abbott, 2009; Kanashiro et al., 2017).

Anatomical data indicates that FEF projections to extrastriate visual cortex (V4) terminate primarily on pyramidal (putatively excitatory) cells (Anderson et al., 2011). In our model we have not explicitly incorporated this asymmetry in top-down projection targets; however, firing rate modulation by top-down input is observed in the inhibitory, rather than excitatory, extrastriate neurons. There are at least two possible ways to resolve this superficial conflict between the existing anatomical data and our model: first, that the relatively small proportion of synapses directly onto inhibitory neurons are nevertheless able to drive these recipient neurons strongly, or second, that inhibitory neurons receive this modulation indirectly through a subpopulation of excitatory cells. Moreover, experimental (Mitchell et al., 2007) and model-based (Kapfer et al., 2007; Vogels and Abbott, 2009; Kanashiro et al., 2017) studies of top-down modulation have consistently identified the importance of preferential activation of inhibitory cells for attention and WM modulation, and the present work is consistent with this literature.

Our modeling results involving WM modulations are at odds with models that postulate supra-linear—i.e., non-saturating—responses in inhibitory networks (Hennequin et al., 2018). In such a situation, when the inhibitory network activity is elevated in a supra-linear fashion, it would produce saturating, not enhancing, excitatory responses, and reduced variability through the stabilizing influence of high-gain inhibitory responses. In our model results, the situation is reversed and we observe saturating inhibition in which gain is higher at low baseline rates, and lower at higher baseline rates. This saturating inhibition enhanced excitatory responses, and also reduced e-cell variability, but only for e-cells that exhibit phase locking. This yields another testable prediction of our model: that spike variability exhibits a dependence on relative frequency between LFP and spike rate, and degree of phase locking of the cell, and is wholly distinct from the variance reduction mechanism proposed in the mean field rate-based model of Hennequin et al. (2018). Note, however, we have not analyzed the role of recurrent excitatory connections as a potential intervening factor, both for effects on variability and also gain modulation.

As stated above, WM-input is associated with enhanced network oscillations in the αβ band, replicating the Power aspect of our experimental results. The emergent network oscillations are due to reciprocal connections within the inhibitory subnetwork, as in numerous computational studies (Van Vreeswijk et al., 1994; Chow et al., 1998; White et al., 1998; Brunel and Hakim, 1999; Whittington et al., 2000; Brunel and Wang, 2001; Lee et al., 2013). In our model, this oscillatory signal shapes the timing of excitatory units' spikes relative to the oscillations, and accounts for the Phase aspect of our results. We further captured both the Gain and Phase aspects of the network model using our phase oscillator model, which confirmed our understanding that inhibitory gain saturation and phase dependence independently contribute to excitatory cell gain and SPL modulation by WM input. Specifically, the phase model explicitly captured the effect of inhibitory input timing on SPL. Inhibition timed to occur just before a cell is nearing spike threshold is more effective than inhibition timed just after spike emission; this is because inhibitory currents rely on ionic currents with membrane reversal potentials below the spiking threshold, and so will be stronger when the cell is closer to threshold. This relationship was captured by a negatively sloped phase response function, representing a phase- or oscillation-dependent modulation of inhibitory input efficacy. This oscillation-dependence greatly enhanced spike phase locking (compared to when the phase response function was not phase-dependent).

Some prior modeling studies use different mechanisms to generate α and β-range oscillations (Spitzer and Haegens, 2017). *In vitro* investigations of somatosensory cortex identified intrinsic bursting triggered through the M-current as a possible driver of faster-frequency β2 rhythms, in which gap-junction coupling is necessary for coherent rhythm generation (Roopun et al., 2006). Such bursting-based oscillatory dynamics have been incorporated into more complex multi-layer models (Kramer et al., 2008; Kopell et al., 2011; Lee et al., 2013). An intralaminar mechanism has been proposed to account for how superficial-layer γ rhythms, arising from fast-spiking interneurons and regular spiking excitatory cells, are interleaved (termed period concatenation) with deep-layer intrinsic bursters that are reciprocally coupled to superficial cells. These burst-based models (Kopell et al., 2011; Lee et al., 2013) have been used to account for a selective WM enhancement effect based on biased competitive inhibition between different oscillator assemblies (Desimone and Duncan, 1995; Humphreys et al., 1998; Ardid et al., 2007). In these burst-based models, the interleaved γ spiking cycles within β cycles are also enhanced by the WM signal. Moreover, β spike phase locking appears in a bi- or tri-modal pattern, where spikes are aligned at two or three phases centered equidistant from crest of the β cycle (see Figure 4C of Kopell et al., 2011). In contrast, our experimental and modeling results show no γ-band LFP enhancement or phase locking, nor γ phase locking modulation by WM. Also, in our model and experimental data, spikes occur in a unimodal distribution of phases in the rising phase of the β cycle, cresting prior to peak inhibition onset (see Figure 1). Thus, the frequency and pattern of SPL modulation observed in our data is better explained by the model presented here.

The model we have developed here possesses reciprocally-connected interneurons mediated by relatively slow-timescale synaptic inhibition (compared to that of GABA_A_, see also Lee et al., 2013). Interneuron activity is enhanced in visual areas during attention tasks (Mitchell et al., 2007); and a modeling study by Jensen et al. (2005) identified a possible role for reciprocally connected interneurons in αβ oscillations via GABA-type inhibitory currents. Here, the dynamic origin of oscillation frequency modulation is inhibitory cell baseline excitability, where spontaneous spiking becomes emergently synchronized (Van Vreeswijk et al., 1994; Chow et al., 1998; White et al., 1998; Brunel and Hakim, 1999; Whittington et al., 2000; Brunel and Wang, 2001). Also, our model is entirely intracolumnar, and the WM or attentional effect does not rely on biased competition between columns that necessarily involve elevated firing rates in the absence of inputs—the opposite of our Parity result—(Desimone and Duncan, 1995; Humphreys et al., 1998; Ardid et al., 2007).

Note also that our model input included extrinsic noise, both broadband white-noise as well as slower-timescale fluctuations, which generated variability in cell behavior. The noise level was tuned to be large enough so that cells in the network exhibited smooth, monotonic firing rate changes in response to input changes rather than stair-step jumps due to broad regions of phase locking stability observed in the low-noise regime (Chacron et al., 2000; Nesse et al., 2007). Here, we assumed the extrinsic noise came from the cortical milieu that was not explicitly modeled; however other modeling investigations have examined networks that self generate chaotic variability without the need for extrinsic noise (Deco et al., 2011; Huang et al., 2019). These studies also establish that activation of inhibitory subnetworks, with slow-timescale inhibitory synapses—either due to synaptic delays, or simply slow rise-times—is a critical mechanism for inducing chaotic network variability. Moreover, attentional modulation also occurs through further activation of inhibitory networks that serve to stabilize network behavior by eliminating slow-timescale chaotic fluctuations between multiple attractors (see also Hennequin et al., 2018 for single attractors). Our model here does not address the source of noise and putative chaotic network fluctuations; however our results are broadly consistent in their emphasis on inhibitory subnetworks as the key underlying modulation that covaries with the attentional or WM signal.

An article by van Elswijk et al. (2010) identifies β-oscillation-based gain modulation arising from the synchronization of many neurons, producing summation in output spikes. While such summation and downstream gain could result from the SPL modulations in our model, we also observe direct modulation of spike rate and variability. The phase-dependent efficacy of inhibition in our model does have great import for coding proposals that rely on the summation of spikes (see also CTC theory, Fries, 2015). Because our model modulates SPL in concert with firing rates, this may serve to enhance the efficacy in driving downstream areas through multiple mechanisms–both increasing the number of spikes, and clustering them in a narrow range of phases of the αβ oscillation (i.e., increased SPL) (Diesmann et al., 1999; Womelsdorf et al., 2007; Kumar et al., 2008, 2010; Fries, 2015; Moldakarimov et al., 2015; Hahn et al., 2019).

## Data Availability Statement

The original contributions presented in the study are included in the article/supplementary material, further inquiries can be directed to the corresponding author: Dr. Behrad Noudoost: behrad.noudoost@utah.edu.

## Ethics Statement

The animal study was reviewed and approved by Montana State University Institutional Animal Care and Use Committee.

## Author Contributions

WN and BN designed experiments. BN collected neural data. ZB analyzed neural data. WN performed the computational modelling. KC, WN, and BN prepared the manuscript.

## Conflict of Interest

The authors declare that the research was conducted in the absence of any commercial or financial relationships that could be construed as a potential conflict of interest.
